# A Study of Comorbidities Among Epilepsy Patients in Selected Hospitals of Khartoum State, Sudan

**DOI:** 10.7759/cureus.88500

**Published:** 2025-07-22

**Authors:** Lina Fadhulalla, Tahir Obeid

**Affiliations:** 1 Medicine and Surgery, University of Medical Sciences and Technology (UMST), Khartoum, SDN; 2 Neurology, University of Medical Sciences and Technology (UMST), Khartoum, SDN

**Keywords:** cognitive, comorbidity, epilepsy, medical, neurological, psychological, sudan

## Abstract

Background: Comorbid conditions in epileptic patients depend on many factors like etiology, type of epileptic syndromes, social and environmental factors, which may differ from one region to another, which encourages us to do this study in Sudan, which is one of the low-income countries. Limited information is available regarding this in our region.

Methods: This is a hospital-based, prospective, descriptive, and cross-sectional study aimed at determining the common medical, neurological, psychological, and cognitive comorbidities. Data were obtained by interviewing 115 epileptic patients attending the outpatient clinic of the Academic Charity Teaching Hospital and the National Center for Neurological Science using a questionnaire from November 2019 to March 2020.

Results: Out of 115 patients, 52.2% (60/115) were females. The duration of epilepsy ranged from 1 year to 37 years. A high rate of unemployment (72.2%) and a low level of education (36.5%) were observed among epileptic patients in Sudan. The number of comorbidities ranged from one (58.3%) to four (0.9%). Neurological comorbidities (67%) were the commonest, followed by medical (32.2%), psychological (26.1%), and lastly cognitive comorbidities (13%). The most prevalent comorbidities were migraine (33.7%), hypertension (27%), depression (50%), and impaired memory (13%).

Conclusion: This study sheds light on important prognostic factors in epileptic patients, which have not been previously analyzed in other research, particularly in Sudan. It introduces a new perspective on the condition, paving the way for further contributions in future studies.

## Introduction

A comorbid condition is the co-occurrence of several medical conditions in the same individual during the course of an index disease (e.g., epilepsy), which can arise at any time before, concomitantly, or after epilepsy diagnosis. It represents distinct clinical diseases and syndromes as signs or symptoms of the principal diagnosis [1,2]. There is a median of two chronic medical conditions generally observed in young adults, and it can rise up to six in patients older than 65 years. Statistically, 50% of adults with active epilepsy have at least one comorbid medical disorder. Comorbidities in epileptic patients are up to eight times more common than in the general population. The treatment of epileptic seizure disorders is not restricted to the achievement of seizure freedom, but must also include the management of comorbid medical, neurological, psychiatric, and cognitive conditions [3,4].

Comorbidities among people with epilepsy are somatic, neurologic, psychiatric, and cognitive. Somatic injuries include physical injuries that can be a direct result of seizures as falls, bone fractures, and other accidents, as well as other medical conditions. Epileptic patients have an increased incidence of cardiovascular, respiratory, inflammatory, and pain disorders, increased rates of heart disease, and high blood pressure. Neurological comorbidities such as migraine, stroke, dementia, cerebral palsy, arteriovenous malformations, brain tumors, cortical malformations, CNS infections, and traumatic brain injury (TBI). Migraines are the most common, with a prevalence of 20-40% [4,5]. Psychiatric comorbidities commonly include mood disorders, anxiety, depression, attention deficit hyperactivity disorder, and psychosis. The prevalence rate is up to 30-35% and depression is the commonest, with an incidence rate of 22% and a lifetime prevalence of around 35% [4,6-8]. Patients with uncontrolled epilepsy may also suffer from depression or anxiety related to the fear of additional seizures, loss of the ability to work, from depressed socioeconomic status, or from the psychotropic effects of anti-seizure drug polytherapy. Cognitive dysfunction is a deficit in learning and problem-solving, memory, academic underachievement, impairments in executive functions and social function, mental speed, attention, and language. It worsens with longer duration of epilepsy, specifically worsening performance in immediate memory, attention, and visual working memory [6,9-11].

Many studies have significantly contributed to identifying the comorbidities associated with epileptic patients. Three key findings from these studies are as follows: A cohort study that was published in 2018 shows that the high prevalence ranking were hypertension (HTN) (19.6%), peripheral vascular disease (8.1%), cardiac arrhythmias (5.8%), dementia (4.6%), renal disease (4.1%), with high rates of comorbidities in the elderly were also noted. An additional case-control study conducted in Sudan on the prevalence of depression among patients with epilepsy shows that 45.5% of the sample were depressed, and mild depression was found in 31.5%. To end with, a study was done in Norway. Nearly 80% of children with epilepsy had more than one comorbid disorder, of which medical disorders were recorded in 55%, neurologic disorders in 41%, and developmental/psychiatric disorders in 43%. This contributes to showing the importance of diagnosis and early detection of the comorbid conditions, as it varies in its presentation [12-14].

## Materials and methods

Study design and setting

This is a prospective, hospital-based, descriptive, cross-sectional study. Data were obtained via a structured questionnaire administered to 133 patients diagnosed with epilepsy during their outpatient clinic visits from the period of November 2019 to March 2020 in selected hospitals in Al-Khartoum state, including the Academic Charity Teaching Hospital (ACTH) and the National Center for Neurological Science (NCNS). The study population was epileptic patients. Inclusion criteria include epileptic patients who received care at the designated hospital from November 2019 to March 2020. Eligible participants must have at least one comorbid condition in addition to epilepsy. Patients who did not meet this criterion were excluded from the study. The questionnaire is included in Appendices A-B.

Sampling technique and sample size

A purposive sampling was used to select the participants. The estimate was calculated using the formula $n = \frac{z^2pq}{d^2}$ for an unknown population. The calculated sample size was 133 epileptic patients; however, due to time constraints, this target was not fully achieved.

Data collection, management, and statistical analysis

Data was collected using a questionnaire from 133 epileptic patients attending the outpatient clinic. Then it was analyzed using IBM SPSS Statistics for Windows, Version 20 (Released 2012; IBM Corp., Armonk, New York, United States) and Microsoft Excel 2010 (Microsoft® Corp., Redmond, WA, USA) for both numerical and graphical representation. Data was presented using descriptive statistics.

Ethical considerations

Approval was obtained from the research technical and ethical committee at the Faculty of Medicine, University of Medical Sciences and Technology (UMST). Written consent from the Ministry of Health was given to start collecting data at the different hospitals where this research was conducted. Informed consent was also obtained from the administrations of ACTH and NCNS. Verbal consent from the patients was sought, where their privacy and confidentiality were maintained.

## Results

This study aims to determine the common medical, neurological, psychological, and cognitive comorbid conditions among epileptic patients.

The mean age of epileptic patients (n=115) was 32.83 years, with the minimum age of onset recorded at one year and the maximum at 82 years. The duration of epilepsy ranged from 1 to 37 years, with a mean duration of 6.14 years. The gender variation of epileptic patients was mostly equal. The female gender (60, 52.2%) exceeded the male gender (55, 47.8%). The age groups of epileptic patients varied: one patient (0.9%) was an infant, five (4.3%) were children, 13 (11.3%) were teenagers, 79 (68.7%) were adults, and 17 (14.8%) were elderly. The age group distribution was statistically significant, with a p-value of 0.009142 (p≤0.05 considered significant).

The majority of epileptic patients included in this study were unemployed (83, 72.2%), while 32 (27.8%) were employed. In terms of level of education, the majority studied until primary school (42, 36.5%), followed by those who studied until high school (27, 23.5%). Illiterate patients were 26 (22.6%), the least two groups were those who graduated from school and entered college (17, 14.8%) and those who are studying in Khalwa (Quran school) (3, 2.6%). This rate shows the burden of comorbid conditions on epileptic patients, ranging from lower education level to higher risk of unemployment.

The majority of patients presented with primary and secondary generalized epilepsy (80, 69.6%). The types of seizures observed among the patients included: tonic-clonic seizures in 66 patients (57.4%), absence seizures in 12 (10.4%), myoclonic seizures in one (0.9%), and atonic seizures in one (0.9%). Additionally, 35 patients (30.4%) presented with focal seizures, which included 15 cases (13%) of focal seizures with awareness and 20 cases (17.4%) without awareness. This distribution was statistically significant, with a p-value of 0.000265.

The anti-seizure medications prescribed to epileptic patients included carbamazepine, which was the most commonly used drug, taken by 64 patients (55.7%). This was followed by sodium valproate in 18 patients (15.7%), levetiracetam in 16 (13.9%), and lamotrigine in five (4.3%). A very small number of patients were prescribed phenytoin or a combination of anti-seizure medications.

Among the different types of comorbid conditions, neurological comorbidities take precedence above all, with an incidence of 83 (72.2%), then medical comorbidities (37, 32.2%), psychological comorbidities (30, 26.1%), and lastly cognitive comorbidities with an incidence of 15 (13%).

There was a range in the number of comorbidities observed among epileptic patients. A total of 67 patients (58.3%) presented with one comorbidity, 33 (28.7%) had two, 14 (12.2%) had three, and one patient (0.9%) presented with four comorbid conditions associated with epilepsy.

Table 1 shows the medical comorbidities, where 10 (27%) had HTN, followed by diabetes mellitus (DM) in five patients (13.5%), cerebral malaria in three (8.1%), and kidney stones and fractures, each in two patients (5.4%). The following comorbidities were each observed in one patient (2.7%): valvular heart disease, rheumatoid arthritis, venous thrombosis and eclampsia, blindness in one eye, thyrotoxicosis, tuberous sclerosis, scorpion bite, acute kidney failure (AKF), sickle cell anemia, tuberculosis (TB), generalized fatigue, urinary tract infection (UTI), irritable bowel syndrome (IBS), myocardial infarction (MI), hemorrhoids, iron deficiency anemia (IDA), supraventricular tachycardia, measles, Wilson’s disease, congenital heart disease (CHD), dizziness, nausea, and end-stage kidney disease (ESKD).

**Table 1:** Incidence of medical comorbidities in epileptic patients HTN: hypertension; DM: diabetes mellitus; AKF: acute kidney failure; TB: tuberculosis; UTI: urinary tract infection; IBS: irritable bowel syndrome; MI: myocardial infarction; CHD: coronary heart disease; ESKD: end-stage kidney disease

	Frequency	Percent (%)
Valvular heart disease	1	2.7
Fractures	2	5.4
Rheumatoid arthritis	1	2.7
Venous thrombosis and eclampsia	1	2.7
Blindness in one eye	1	2.7
Thyrotoxicosis	1	2.7
Tuberous sclerosis	1	2.7
HTN	10	27
scorpion bite	1	2.7
Cerebral malaria	3	8.1
DM	5	13.5
AKF	1	2.7
Kidney stones	2	5.4
Sickle cell anemia	1	2.7
TB	1	2.7
Generalized fatigue	1	2.7
UTI	1	2.7
IBS	1	2.7
MI	1	2.7
Hemorrhoids	1	2.7
Iron deficiency anemia	1	2.7
Supraventricular tachycardia	1	2.7
Measles	1	2.7
Wilson's disease	1	2.7
CHD	1	2.7
Dizziness	1	2.7
Nausea	1	2.7
ESKD	1	2.7

The time of diagnosis of the medical comorbid conditions helped to identify the possible cause of association. A total of 27 (73%) patients presented with it before being diagnosed with epilepsy, while 11 (29.7%) presented after the diagnosis. In term of possible causes of medical comorbidities, 25 (67.6%) showed no possible association, in nine patients (24.3%), the comorbid condition was the primary cause, in three patients (8.1%), it was resultant from epilepsy and in other three patients (8.1%), the cause was a side effect from anti-seizure medications, with a p-value of 0.059988 (p≤0.05 considered significant).

Table 2 shows the neurological comorbidities observed among epileptic patients. The most common condition was migraine, reported in 28 patients (33.7%), followed by head trauma in 15 (18.1%), and stroke in 13 (15.7%). Additional comorbidities included lower limb weakness in four patients (4.8%), meningitis in three patients (3.6%), cerebral palsy in three patients (3.6%), mental retardation in two patients (2.4%), muscle spasms in two patients (2.4%), skull fractures in two patients (2.4%), brain hemorrhage in two patients (2.4%), and tremors in two patients (2.4%). Several conditions were each reported in one patient (1.2%), including post-traumatic hemiplegia, left hemi-facial spasm, brain abscess, cerebellar ataxia, dementia, infantile hemiplegia, sciatica, dystonia, unilateral deafness, amnesia, Parkinson’s disease, subdural hematoma, and viral encephalitis.

**Table 2:** Incidence of neurological comorbidities in epileptic patients LL weakness: lower limb weakness

	Frequency	Percent (%)
Migraine	28	33.7
Mental retardation	2	2.4
Head trauma	15	18.1
Muscle spasm of the left leg	2	2.4
Post-traumatic hemiplegia	1	1.2
Stroke	13	15.7
Skull fracture	2	2.4
Left hemifacial spasm	1	1.2
LL weakness	4	4.8
Meningitis	3	3.6
Brain hemorrhage	2	2.4
Brain abscess	1	1.2
Cerebellar ataxia	1	1.2
Cerebral palsy	3	3.6
Dementia	1	1.2
Infantile hemiplegia	1	1.2
Tremors	2	2.4
Sciatica	1	1.2
Dystonia	1	1.2
Deafness in the left ear	1	1.2
Amnesia	1	1.2
Parkinson's disease	1	1.2
Subdural hematoma	1	1.2
Viral encephalitis	1	1.2

The timing of diagnosis for neurological comorbidities helped show that 46 patients (59.7%) presented before being diagnosed with epilepsy, while 43 patients (55.8%) presented after being diagnosed, with a p-value of 0.0513170 (p ≤ 0.05 considered significant). Regarding the possible causes of association, in most cases, the comorbidity was identified as the primary cause of epilepsy (42 patients, 54.5%), followed by cases where the condition resulted from epilepsy (39 patients, 50.6%). In 10 patients (12%), no clear association could be determined.

Table 3 presents the psychological comorbidities observed among epilepsy patients. Depression was associated with 15 patients (50%), followed by anxiety in seven patients (23.3%), and abnormal behavior in four patients (13.3%). Attention-deficit hyperactivity disorder (ADHD) was observed in two patients (6.7%). Additionally, one patient (3.3%) was diagnosed with autism spectrum disorder, and another (3.3%) with postpartum psychosis.

**Table 3:** Incidence of psychological comorbidities in epileptic patients ADHD: attention deficit hyperactivity disorder

	Frequency	Percent (%)
Depression	15	50
Autistic spectrum disorder	1	3.3
ADHD	2	6.7
Abnormal behavior	4	13.3
Anxiety	7	23.3
Postpartum psychosis	1	3.3

Regarding the time of onset, psychological comorbidities were reported after the diagnosis of epilepsy in 28 patients (93.3%), while only two patients (6.7%) presented before epilepsy.

Regarding the possible cause of association, psychological comorbidities were attributed to epilepsy in 27 patients (90%). In two cases (6.7%), the comorbid condition was identified as the primary cause of epilepsy, while in one case (3.3%), no clear association could be determined.

The cognitive dysfunction among patients mainly presented with impaired memory (15, 13%). All 15 patients (100%) were diagnosed with the cognitive comorbid condition after the onset of epilepsy, and in all cases, the condition was concluded to be caused by epilepsy.

The statistical analysis examining the associations between the types of epilepsy (focal, primary generalized, and secondary generalized) and the comorbidities (medical, neurological, psychological, and cognitive) showed no significant associations, except for meningitis, which demonstrated a significant association (p-value = 0.028). A p-value less than 0.05 indicates a statistically significant association.

With regard to the association between the number of comorbidities and the duration of epilepsy, the results showed a weak positive correlation. Similarly, a weak positive correlation was found between age and the number of comorbidities. The Pearson correlation coefficients were 0.239 and 0.135, respectively. Correlation is considered significant at the 0.05 level.

## Discussion

Comorbidities heavily impact the quality of life in epilepsy patients, where there is a high degree of unemployment (72.2%) and a low level of education (36.5%). It highlights disparities between healthcare systems in high-income countries and low-income ones like Sudan, where resources and support are scarce. This aligns with the observations of Gutiérrez-Ángel et al., who emphasized the burden of comorbid conditions in epilepsy patients [15].

Our major results are provided as follows: medical comorbidities with the highest incidence were HTN (27%) and DM (13.5%). Neurological comorbidities appeared with the highest overall incidence, commonly including migraine (33.7%), head trauma (18.1%), and stroke (15.7%). Psychological comorbidities showed a high incidence of depression (50%). Cognitive dysfunction was observed in all cases as impaired memory.

The results of our study provide further evidence supporting the theories presented in previous literature about comorbidities and epilepsy. A previous review article done in China in 2018 showed that the prevalence of comorbidities was HTN (19.6%) [12]. Similarly, a study conducted in Thailand in 2019 found that the prevalence of depressive disorders was the highest (17.1%) [16]. In addition, a study carried out in Kenya reported a strong association between epilepsy and cognitive impairments (odds ratio (OR) = 14.55, 95% confidence interval (CI) = 3.52-60.14) [5].

The presence of conflicting findings regarding comorbidities in different studies highlights the variability in results observed across various countries, which can be attributed to a multitude of factors. This underscores the necessity of conducting in-depth investigations into each specific type of comorbidity to enhance our understanding of epilepsy.

There were some limitations to this study. First, confirmation of the relationship between comorbidities and epilepsy is not available due to a lack of facilities responsible for such investigations, and this highly selective group does not represent the true general population. Furthermore, the continued use of the old classification system for epilepsy may affect the results, as newer classification standards were not applied.

Based on the findings of this study, we recommend further research addressing epilepsy and its comorbidities, with future insights aimed at improving the quality of patients' lives.

## Conclusions

In conclusion, this study sheds light on important prognostic factors in epileptic patients, which have not been previously analyzed in other research, particularly in Sudan. It introduces a new perspective on the condition, paving the way for further contributions in future studies.

## Figures and Tables

**Figure 1:**
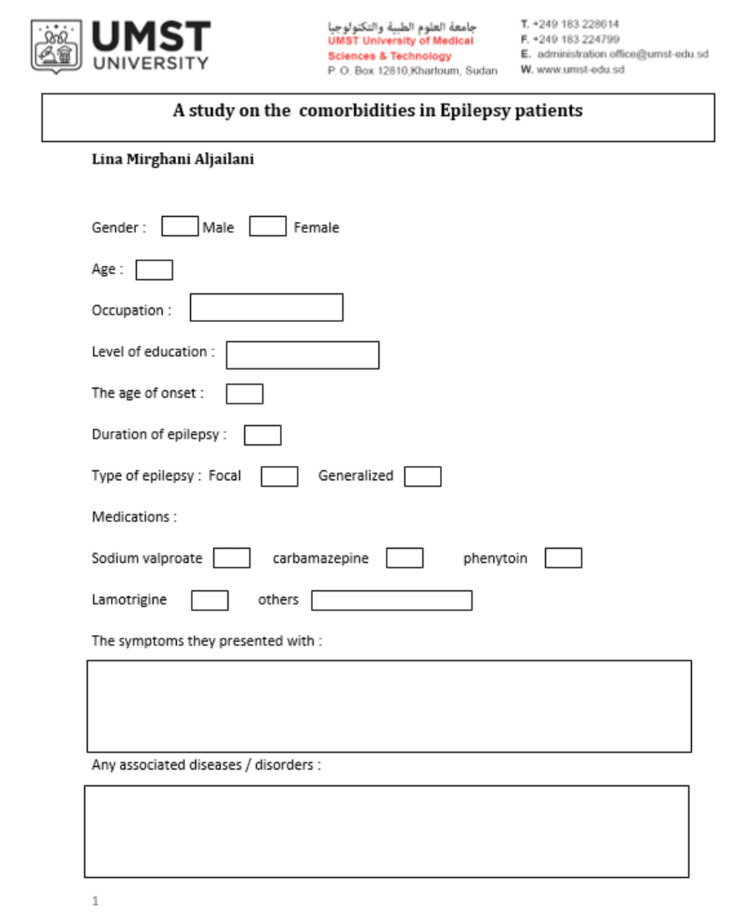
A structured questionnaire designed to identify common comorbid conditions in patients with epilepsy attending the outpatient clinic. It focuses on collecting relevant data and enhance comprehensive understanding of the condition. Page 1 of the questionnaire. This questionnaire was developed by the authors.

**Figure 2:**
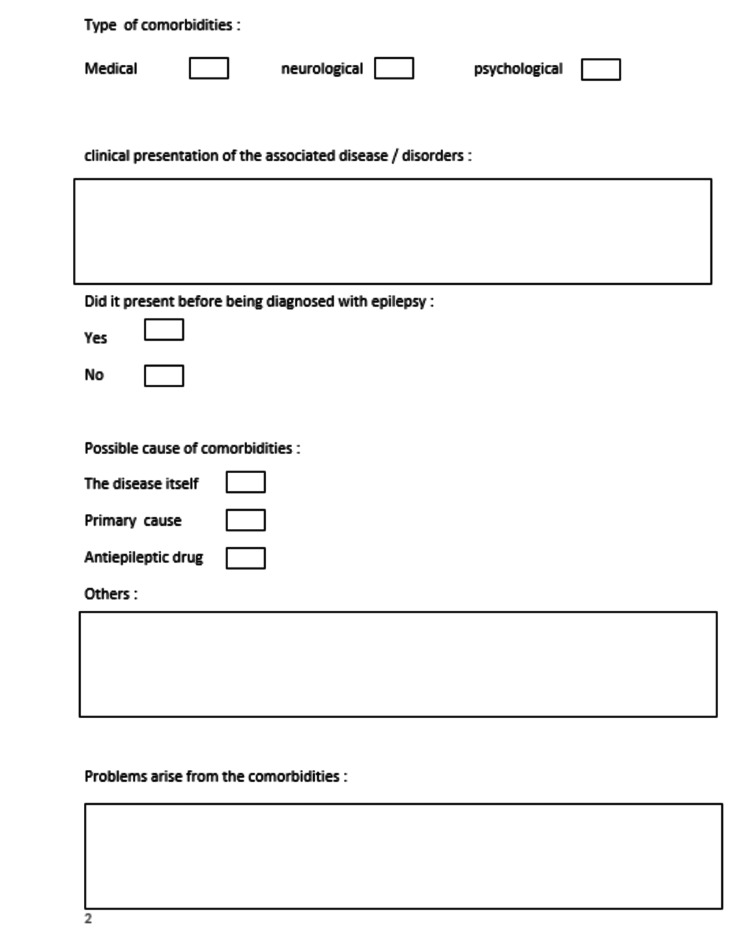
Page 2 of the questionnaire. This questionnaire was developed by the authors.

## Appendices

Appendix A 

Appendix B 
